# Profiles of PhD students’ satisfaction and their relationships with demographic characteristics and academic career enthusiasm

**DOI:** 10.3389/fpsyg.2022.968541

**Published:** 2022-10-27

**Authors:** Yang Yang, Jianqiao Cai

**Affiliations:** School of Educational Science, Hunan Normal University, Changsha, China

**Keywords:** PhD student’, satisfaction, academic career enthusiasm, person-centered approach, two-factor theory, latent profile analysis

## Abstract

The satisfaction of doctoral students is very important for the quality of higher education. Based on two-factor theory (also known as Herzberg’s motivation–hygiene theory), this study used a person-centered approach to examine possible doctoral student satisfaction profiles. In total, 4,964 participants were included in the study, and the results of latent profile analysis showed that they could be classified into four subgroups: (i) the low-motivation–low-hygiene group (700 participants, 14.1% of the sample), (ii) the low-motivation–high-hygiene group (979, 19.7%), (iii) the high-motivation–low-hygiene group (1,554, 31.3%), and (iv) the high-motivation–high-hygiene group (1,731, 34.9%). Analyses showed that the PhD students differed significantly in their satisfaction-profile membership depending on their gender, age, country, study-abroad status, work status, and caring responsibilities. Specifically, male students, younger students, and students studying abroad tended to be more satisfied with both motivation and hygiene factors. Besides, regarding maintaining and stimulating doctoral students’ academic career enthusiasm, motivation factors can compensate for the negative impact of the absence of hygiene factors, not the other way around. Therefore, it can be seen that two-factor theory has a certain explanatory power for changes in academic career enthusiasm, but it must be adjusted in a certain way considering the special characteristics of the population.

## Introduction

Doctoral education is experiencing rapid expansion worldwide (Gruzdev et al., 2020) and is becoming an increasingly important factor in driving socioeconomic development (Dericks et al., 2019). In this context, the quality of doctoral education is of widespread concern to both governments and society (Byrne et al., 2013). As the key subjects of doctoral education, the feelings of PhD students about the training process are regarded as an important indicator for evaluating the quality of doctoral education. Setting performance indicators is a management tool to guarantee the quality of higher education, but its market-oriented nature inevitably leads to the commodification of higher education and the consequent transformation of students into “consumers” (Naidoo and Williams, 2014). A study of six European countries partially supports this view, pointing to an increased tendency toward consumerism in higher education policy and practice (Brooks, 2022). It can be concluded that to some extent, PhD students are to doctoral education as consumers are to various other products (Marzo-Navarro et al., 2005). Therefore, as a special commodity, the quality of doctoral education is closely related to the satisfaction of doctoral students with the training process (Cheng et al., 2016). In this context, PhD students’ satisfaction (PhD-SS) has been regarded as an effective means of assessing and promoting the quality of doctoral education (Barnes and Randall, 2012). PhD-SS can be defined broadly as feelings or perceptions that are used to express PhD students’ responses to whether the doctoral training process meets their expectations (Hartman and Schmidt, 1995; Rowley, 1996; Munteanu et al., 2010; Kahu, 2013). Previous studies of PhD-SS have found that satisfaction with the training process can improve academic performance and contribute to positive organizational behavior (Pike, 1993; Sung and Yang, 2009; Gibbons et al., 2015). Based on that previous research, we can reasonably infer that PhD-SS may inspire PhD students to identify with and be passionate about their academic research careers (Dericks et al., 2019).

Although less abundant than studies on undergraduate student satisfaction, those on PhD-SS have produced some valuable research results, particularly in two main aspects. First, much research effort has gone into providing empirical evidence for the causal relationship between PhD-SS and its outcomes, including positive outcomes (e.g., retention, success) (Fairbanks, 2016; van Rooij et al., 2021) and negative outcomes (e.g., attrition, delay, mental-health problems) (Golde, 2005; Pyhältö et al., 2009). Another body of research on PhD-SS has been focused on identifying its composition and determinants (Dericks et al., 2019; Kulikowski et al., 2019), and most of those studies generally suggested that supervisors play a vital role in PhD-SS (Erichsen et al., 2014; Boyce et al., 2019; Gruzdev et al., 2020). Besides, course quality, team climate, financial support, and future job prospects have also been confirmed to be closely correlated with PhD-SS (Shapiro et al., 2017; Shin et al., 2018). However, those previous studies on PhD-SS relied mainly on a variable-centered approach assuming that all samples were perfectly homogeneous, which is far from reality (Hofmans et al., 2020). Admittedly, the variable-centered approach is useful for examining the relationship between PhD-SS and its antecedents and outcomes, but it misses another core element of PhD-SS, i.e., the PhD students themselves. In other words, satisfaction is an individual’s active perception, and the same factor in exactly the same situation may contribute to different levels of satisfaction in different populations. This is because the individual’s background, experience, and other personal factors might interact with the external factors to form a “satisfaction” judgment.

Indeed, “satisfaction” consists of various elements that may contradict each other; for example, something that helps students to develop intellectually may be a dissatisfied experience emotionally (Collini, 2012). Therefore, PhD-SS should be understood as a complex process. However, the existing research on PhD-SS has been overly focused on how well institutions and especially supervisors help doctoral students’ success (Cheng et al., 2016), trying to study PhD-SS from a variable-centered perspective. However, few studies have acknowledged the heterogeneity in the doctoral student population, meaning that individual differences have been ignored for a long time, thereby leaving this area largely understudied. To fill this research gap, we adopted a person-centered approach in the form of latent profile analysis (LPA). Using the *NATURE PhD SURVEY 2019* dataset, we identified possible profiles of PhD-SS in the training process and explored the related demographics (i.e., gender, age, country, work status, caring responsibilities) that may be the antecedents of the different profiles. In addition to identifying satisfaction profiles, we explored career development process by comparing changes in academic career enthusiasm (ACE) (i.e., decrease, no change, and increase).

## Theoretical framework

Developed by Herzberg et al. (1993), two-factor theory is also known as motivation–hygiene theory. According to this theory, the factors that influence the performance of employees can be divided into two categories: (i) motivation factors, which are necessary for individuals’ professional growth and self-actualization, leading to positive behavior and attitudes to work when people feel satisfied with these factors, and (ii) hygiene factors, which produce no motivation effects even if satisfied, resulting in negative behavior when people feel unsatisfied with them. Two-factor theory is considered as a breakthrough of Maslow’s hierarchy of needs theory and is applied widely in job-satisfaction research (Dion, 2006). Scholars have extended this theory, applying it to research on higher education. Some of them have taken the university student population as their subject, investigating the study motivators and engagement of college students and the persistence of STEM students under two-factor theory (Rizkallah and Seitz, 2017; Gibbs and Wood, 2021; Pedraza and Chen, 2021). The others have paid more attention to teachers working in higher-education institutes. By using Herzberg’s two-factor theory, they have explored lecturers’ motivations to teach (Bett, 2019) and to take up teaching as a career (Amoako et al., 2020), while Marasi et al. (2022) sought the determining factors influencing teachers’ satisfaction with online teaching. Based on previous studies, we propose Herzberg’s two-factor theory may serve as a useful conceptual framework to help us identify potential categories of PhD-SS.

According to the two-factor theoretical framework, the factors that may influence a doctoral student’s perception of a doctoral program can be summarized into two parts. One is motivation factors, which comes from the attraction of academic research itself, leading to a sense of achievement from academic work. The other is hygiene factors, which comes from the external environment (DeShields et al., 2005), leading to a negative feeling of disgust or resistance to academic career when a PhD student is dissatisfied with those factors. It is almost impossible for every PhD student to be satisfied with all of the above elements of the PhD training process (Collini, 2012). Thus, we assumed that PhD students can be classified into four potential categories based on their satisfaction with motivation and hygiene factors.

Scholars have explored the relationship between demographic characteristics and PhD-SS. Existing studies have shown that satisfaction with doctoral study among female doctoral students is significantly lower than that of men (van Rooij et al., 2021; Wang et al., 2021). A significant difference in satisfaction was also found among students of different nationalities (van Rooij et al., 2021). In addition, Harman (2003) found that international doctoral students were more satisfied overall than were national ones, which was supported by a study from Denmark (Kolmos et al., 2008). Besides, scholars have suggested that doctoral students with parenting responsibilities are more likely to face mental health problems (Levecque et al., 2017), which may impact their satisfaction. Considering that doctoral students of different age and work status may have different expectations of doctoral programs, it is reasonable to suspect that PhD-SS also differs in these two characteristics. Thus, we assumed that sociodemographic characteristics (i.e., gender, age, country, study-abroad status, work status, caring responsibilities) are associated with PhD-SS latent class membership.

ACE refers to the intention and interest of doctoral students in pursuing academic research as a career. The PhD program is an important training phase for doctoral students toward an academic career, where young students become closely connected to academic work. Therefore, satisfaction at this stage may be highly relevant to their eventual career choices. Given the positive association between PhD-SS and ACE (Dericks et al., 2019; van Tienoven et al., 2022), we assumed that the change of students’ ACE differs as a function of latent class membership.

## The present research

The objective of this study is to investigate the profiles of PhD-SS and its association with demographic variables and the changes in ACE. According to previous study, three hypotheses were proposed, as follows:

Hypothesis 1: *PhD students can be classified into four potential categories based on their satisfaction with motivation and hygiene factors*.

Hypothesis 2: *Gender, age, country, study-abroad status, work status, and caring responsibilities are associated with PhD-SS latent class membership*.

Hypothesis 3: *PhD-SS profiles are associated with the changes in ACE*.

## Materials and methods

### Participants

The data were selected from the questionnaire responses of a global survey of PhD students (Nature Research, 2019) with a total of 6,812 valid cases. Given that this dataset contains information from all over the world and on various topics related to PhD students, there is no doubt about its representativeness (Li and Horta, 2021). Data on responses to two scales (reported in the Measures Section) were included in this analysis. The data were cleaned according to a seven-point scoring system, and those samples for which either participants failed to respond or the answers were outside the range of 1–7 were excluded from the analysis, leading to a final sample of 4,964 participants. This number of observations is large enough to ensure a good level of statistical power. Among the valid samples, most were studying in Europe (1,677 participants, 33.78% of the sample), Asia (1,469, 29.59%), North America (1,404, 28.28%), and others in Africa, Australasia and South America (414, 8.35%); more specifically, the largest number of cases came from the United States (1,162, 23.41%), followed by China (673, 13.56%), India (398, 8.02%), Germany (341, 6.87%), the United Kingdom (334, 6.73%), and cases from other countries (2056, 41.42%). The gender ratio of those observations was near 1:1, though there are some disparities in gender ratios between countries (i.e., In China, female:male = 0.52:1; in UK, female:male = 1.53:1; in USA, female:male = 1.34:1; in Germany, female:male = 1:1). Also, most cases were between the ages of 25 and 34.

### Measures

#### PhD student satisfaction

To measure PhD-SS, a question that included 18 items was used, namely, “How satisfied are you with each of the following attributes or aspects of your PhD?.” The respondents rated the extent to which they agreed with those 18 items using a seven-point scale (reported in Table 1), with higher values indicating higher satisfaction. Exploratory factor analysis (EFA) was conducted to establish the essential structure of those 18 items and synthesize them into a few core factors, and in this way four factors with eigenvalues greater than one were extracted. Factor 1 contained eight items, such as “recognition from supervisor/PI” and “number of publications”; based on its common characteristics, we named it *satisfaction with academic cultivation* (SAC). Factor 2 contained the two items of “ability to attend meetings and conferences” and “ability to present research at conferences”; we named it *satisfaction with academic interaction* (SAI). Factor 3 contained five items, such as “work–life balance,” “vacation time,” and “social environment”; we named it *satisfaction with academic life* (SAL). Factor 4 contained three items, such as “availability of funding” and “stipend financial support”; we named it s*atisfaction with economics* (SWE). Based on the two-factor theoretical framework, we regard factors 1 and 2 as being motivation factors because they are closely related to the academic research itself, while we regard factors 3 and 4 as being hygiene factors because they are focused more on the external environment, especially on the lives of doctoral students.

**Table 1:** Results of exploratory factor analysis.

	SAC	SAL	SAI	SWE
Recognition from supervisor/PI	0.842			
Overall relationship with supervisor/PI	0.836			
Guidance received from adviser in lab/research	0.818			
Guidance received from other mentors in lab/research	0.665			
Opportunities to collaborate	0.595			
Career pathway guidance and advice	0.589			
Degree of independence	0.535			
Number of publications	0.427			
Work-life balance		0.778		
Vacation time		0.757		
Hours worked		0.753		
Benefits (health care, leave, etc.)		0.528		
Social environment		0.474		
Ability to present research at conferences			0.895	
Ability to attend meetings and conferences			0.876	
Availability of funding				0.822
Stipend/financial support				0.82
Teaching duties				0.346

SAC, satisfaction with academic cultivation; SAI, satisfaction with academic interaction; SAL, satisfaction with academic life; SWE, satisfaction with economics.

#### Academic career enthusiasm

To measure the changes in ACE of the PhD students, we selected the question “How much more likely are you now to pursue a research career than when you launched your PhD program?” The changes were estimated by means of five options: (i) “equally likely” indicates unchanged ACE during the PhD program, (ii) “much less likely” and (iii) “somewhat less likely” indicate a large decrease and a small decrease, respectively, while (iv) “somewhat more likely” and (v) “much more likely” indicate a small increase and a large increase, respectively.

#### Demographic variables

General information about the PhD students was reported in this survey, including gender, age, country, study-abroad status, work status, and caring responsibilities.

### Statistical analysis

The statistical analysis involved three stages. In the first stage, some preliminary analyses were conducted using Excel and SPSS: Excel was used to exclude cases with missing values, while SPSS 21.0 was used to conduct descriptive statistical analysis and EFA. In the second stage, LPA was conducted to extract PhD-SS profiles using Mplus 8.3: we started with two profiles and then added one more each time, stopping when the fit indices (LMR and BLRT) were no longer significant; other fit indices including AIC, aBIC, and Entropy were used to select the best-fitting model. In the third stage, antecedents and consequences of satisfaction were examined using SPSS 21.0; multiple logistic regression was conducted to explore how the satisfaction profiles differ by demographic variables, while a Chi-square test was conducted to compare the retained profiles’ differences in changes of ACE.

## Results

### Common method bias analysis

Because the data were collected in a self-reported questionnaire, common method bias was a possibility. Therefore, we used Harman single-factor inspection (Zhou and Long, 2004) to examine this possible problem before data analysis. The results showed KMO = 0.877 (*p* < 0.001); four common factors with eigenvalues greater than one were extracted, with the first factor accounting for 36.60% of the variance. Therefore, there was no serious common method bias in this study.

### Descriptive statistics and bivariate correlations

Table 2 gives the means, standard deviations, and Pearson correlation coefficients. The means indicate that there is still room for improvement in both the satisfaction and ACE of doctoral students. In addition, PhD students’ ACE is positively associated with their satisfaction, as well as with each dimension of satisfaction.

**Table 2:** Descriptives and correlations (*N* = 4,964).

	M ± SD	1	2	3	4	5
1. Overall	4.46 ± 1.06	1				
2. SAL	4.23 ± 1.32	0.80**	1			
3. SAI	4.93 ± 1.66	0.66**	0.38**	1		
4. SAC	4.52 ± 1.29	0.88**	0.52**	0.51**	1	
5. SWE	4.40 ± 1.39	0.65**	0.52**	0.35**	0.35**	1
6. ACE	3.26 ± 1.25	0.28**	0.18**	0.17**	0.33**	0.05**

**p < 0.01; M ranges from 1 to 5.

### Latent profile analysis of PhD students’ satisfaction

The 18 items of the satisfaction scale were included as indicators to conduct LPA, and the fit indices are given in Table 3. As can be seen, the value of LMR is not significant (*p* = 0.153) when continuing to seven subgroups, indicating that the seven-profile model is not better than the six-profile one (Berlin et al., 2014). Further comparison of the models with two to six profiles shows that the values of AIC, BIC, and aBIC decrease with increasing number of profiles (the lower those fit indices, the better the model fit). However, the decrease becomes slighter between the four-profile and five-profile solutions, and the four-profile one has excellent classification accuracy with a high entropy value (Spurk et al., 2020) of 0.862, which is higher than those of the five-profile (0.853) and six-profile (0.850) solutions. Considering the principle of parsimony, the four-profile model is an interesting alternative. Furthermore, the average class probabilities of the four subcategories range from 0.89 to 0.95, indicating that the classification results of each category are reliable. In summary, the four-profile solution was retained as the best model of PhD-SS (Table 4).

**Table 3:** Model fits for optimal number of profiles in latent profile analysis.

Model	AIC	BIC	aBIC	Entropy	LMR(p)	BLRT(p)	Profile: P	LCP
2C	339216.09	339574.14	339399.36	0.902	0.000	0.000	C1: 0.39	0.96
							C2: 0.61	0.98
3C	334035.06	334516.80	334281.65	0.856	0.000	0.000	C1: 0.43	0.92
							C2: 0.22	0.95
							C3: 0.35	0.94
4C	330778.02	331383.44	331087.92	**0.862**	0.000	0.000	C1: 0.14	0.94
							C2: 0.20	0.89
							C3: 0.31	0.90
							C4: 0.35	0.95
5C	328778.20	329507.32	329151.42	0.853	0.000	0.000	C1: 0.12	0.94
							C2: 0.18	0.89
							C3: 0.13	0.88
							C4: 0.32	0.89
							C5: 0.25	0.92
6C	327392.86	328245.66	327829.39	0.850	0.000	0.000	C1: 0.07	0.92
							C2: 0.12	0.90
							C3: 0.17	0.85
							C4: 0.14	0.89
							C5: 0.26	0.88
							C6: 0.25	0.92
7C	326199.79	327176.28	326699.64	0.853	0.153	0.000	C1: 0.07	0.93
							C2: 0.11	0.82
							C3: 0.10	0.90
							C4: 0.12	0.88
							C5: 0.20	0.87
							C6: 0.14	0.86
							C7: 0.25	0.93

*N* = 4,964; AIC, Akaike information criterion; BIC, Bayesian information criterion; SaBIC, sample-adjusted Bayesian information criterion; LMR(*p*), value of *p* for adjusted Lo–Mendell–Rubin test; BLRT(*p*), value of *p* for bootstrapped likelihood ratio test; LCP, average latent class probability for most likely latent class membership.

**Table 4:** Description of latent profiles (*N* = 4,964).

Profiles	SAC	SAI	SAL	SWE
Low–low	2.46 ± 0.71	3.00 ± 1.65	2.66 ± 0.96	3.28 ± 1.37
Low–high	3.59 ± 0.63	4.97 ± 1.36	4.39 ± 0.93	4.65 ± 1.16
High–low	4.69 ± 0.66	4.59 ± 1.52	3.55 ± 0.94	3.72 ± 1.16
High–high	5.69 ± 0.62	5.98 ± 1.04	5.38 ± 0.83	5.29 ± 1.07
*F*	4964.99***	843.84***	1943.12***	751.70***
Post-hoc	1 < 2 < 3 < 4	1 < 3 < 2 < 4	1 < 3 < 2 < 4	1 < 3 < 2 < 4

***p < 0.001; SAC, satisfaction with academic cultivation; SAI, satisfaction with academic interaction; SAL, satisfaction with academic life; SWE, satisfaction with economics. SAC and SAI referred to “motivation factors;” SAL and SWE referred to “hygiene factors.”

**Figure 1:**
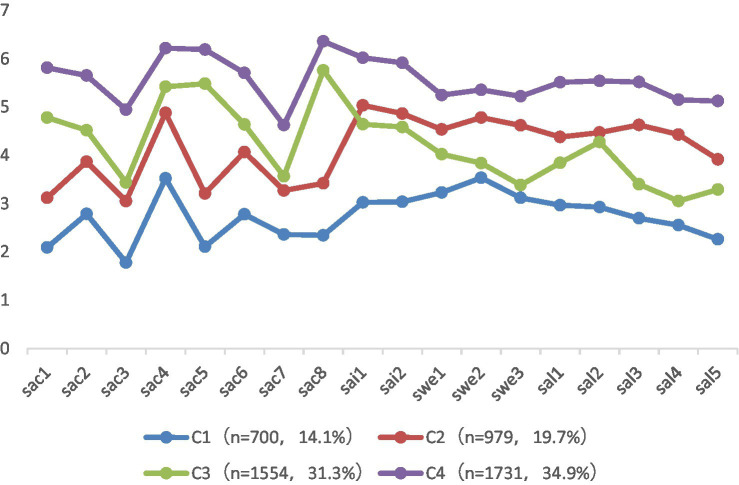
Mean scores of indicators for four-profile solution (*N* = 4,964). sac = satisfaction with academic cultivation; sai = satisfaction with academic interaction; swe = satisfaction with economics; sal = satisfaction with academic life.

Figure 1 shows the means of the 18 satisfaction indicators for the four-profile model. The four identified profiles are interpreted based on this two-factor theoretical framework. Profile 1 (*n* = 700, 14.1% of the sample) shows the lowest values in all four dimensions of PhD-SS, so it is named the *low-motivation–low-hygiene* group. Profile 2 (979, 19.7%) shows a slightly higher value of SAC than that of profile 1 but lower than those of profiles 3 and 4, and it shows higher values of SAL and SWE than those of profile 3 but lower than those of profile 4, so it is named the *low-motivation–high-hygiene* group. The trend of the line graph for profile 3 (1,554, 31.3%) is opposite to that for profile 2, so we name the former the *high-motivation–low-hygiene* group. Profile 4 (1,731, 34.9%) shows the highest values in all four dimensions, so it is named the *high-motivation–high-hygiene* group and is the largest of the four subgroups.

According to the results of the one-way analysis of variance, these four profiles show significant differences in SAC (*F* = 4694.99, *p* < 0.001), SAI (*F* = 843.84, *p* < 0.001), SAL (*F* = 1943.12, *p* < 0.001), and SWE (*F* = 751.70, *p* < 0.001). In addition, the post-hoc test results show that significant differences are found for all possible pair-wise comparisons.

### Differences between latent profiles in demographic variables

In this step of the analysis, logistic regression analysis was conducted to explore the effects of demographic variables on satisfaction. Because a single multinomial logistic model can only compare one group with three other groups, which does not allow for a two-by-two comparison of all types, a binomial logistic regression model is more applicable in this study. The logistic regression was conducted six times so that the results included all pair-wise comparisons, as given in Table 5.

**Table 5:** Association between profiles and demographic variables.

	Reference	Reference compared with	C1 vs. C2		C1 vs. C3		C1 vs. C4	
			*b*	*OR*	*b*	*OR*	*b*	*OR*
Gender	Male	Female	0.03	1.03	−0.22*	0.81	−0.43***	0.65
Study abroad	Yes	No	0.15	1.16	0.33**	1.39	−0.01	0.99
Age	35 and above	18–24	0.38	1.47	0.41	1.5	0.67**	1.96
		25–34	0.36	1.43	0.06	1.06	0.02	1.02
Country	China	USA	0.40*	1.49	0.25	1.28	0.85***	2.34
		UK	0.39	1.48	0.07	1.07	0.61**	1.83
		Germany	0.67**	1.95	−0.44	0.65	0.80***	2.21
		India	−0.12	0.89	0.28	1.33	0.4	1.49
		Others	0.37*	1.45	0.35*	1.43	0.83***	2.29
Job	Yes	No	−0.11	0.89	−0.25*	0.78	−0.08	0.93
Caring	Yes	No	0.55***	1.73	0.2	1.22	0.17	1.19
	Reference	Reference compared with	C2 vs. C3		C2 vs. C4		C3 vs. C4	
			*b*	*OR*	*b*	*OR*	*b*	*OR*
Gender	Male	Female	−0.24**	0.79	−0.45***	0.64	−0.21**	0.81
Study abroad	Yes	No	0.19*	1.21	−0.15	0.86	−0.34***	0.71
Age	35 and above	18–24	0.02	1.03	0.29	1.34	0.27	1.31
		25–34	−0.30	0.74	−0.34*	0.71	−0.04	0.96
Country	China	USA	−0.15	0.86	0.45**	1.57	0.60***	1.83
		UK	−0.32	0.73	0.22	1.24	0.54**	1.71
		Germany	−1.10***	0.33	0.13	1.14	1.23***	3.43
		India	0.40*	1.5	0.52**	1.68	0.12	1.12
		Others	−0.02	0.98	0.46***	1.6	0.48***	1.61
Job	Yes	No	−0.14	0.87	0.04	1.04	0.18	1.19
Caring	Yes	No	−0.35**	0.71	−0.37**	0.69	−0.02	0.98

*N* = 4,740; OR, odds ratio; the profile before “vs.” is the reference group for multiple regression. *p < 0.05; **p < 0.01; ***p < 0.001.

The results show that gender, study-abroad status, age, country, work status, and caring responsibilities all contribute to predicting the PhD-SS profile. Specifically, women were less likely to be classified in the high-motivation–low-hygiene and high-motivation–high-hygiene groups but not the low-motivation–low-hygiene group, while PhD students who studied in their home countries were more likely to be classified in the high-motivation–low-hygiene group than in the low-motivation–low-hygiene and low-motivation–high-hygiene groups. Also, there was no continuity in the differences between age groups, as evidenced by the fact that compared to PhD students older than 35, those aged 18–24 were more likely to be classified in the high-motivation–high-hygiene group than in the low-motivation–low-hygiene group, while those aged 25–34 were more likely to be classified in the low-motivation–high-hygiene group than in the high-motivation–high-hygiene group. Then, compared to PhD students studying in China, those studying in the US, UK, and Germany were more likely to be classified in the high-motivation–high-hygiene group than in the low-motivation–low-hygiene group. Additionally, PhD students who had a job alongside their studies were more likely to be classified in the low-motivation–low-hygiene group than in the high-motivation–low-hygiene group, while PhD students without caring responsibilities were more likely to be classified in the low-motivation–high-hygiene group than in the others.

### Relationship between academic career enthusiasm and PhD students’ satisfaction profile

Table 6 displays the results of comparing the four profiles in terms of changes in ACE. The results of the Chi-square tests show that the changes differed significantly across the four profiles (*p* < 0.001). On one hand, the results of horizontal comparison find that the proportion of PhD students who experienced a decline (slight or serious) in ACE decreased with increasing satisfaction, with 48.1% of samples in the low-motivation–low-hygiene group and only 15.5% in the high-motivation–high-hygiene group; of these, only 3.8% of PhD students in the high-motivation–high-hygiene group had a serious decrease in ACE. Conversely, the proportion of PhD students who experienced an increase (slight or sharp) in ACE increased with increasing satisfaction, with more than 50% of samples in the high-motivation–high-hygiene group and *ca.* 25% in the low-motivation–low-hygiene group. Besides, the percentage of PhD students with no change in ACE was around 30% in all profiles. On the other hand, some interesting results were found from a vertical perspective. First, in the low-motivation–high-hygiene group, the ratio of decreasing, unchanged, and increasing ACE was close to 1:1:1. Then, in the high-motivation–high-hygiene group, the number of students with increasing ACE was more than three times that with decreasing ACE. Also, there was a mirroring characteristic of the ratio of increasing and decreasing ACE between the low-motivation–low-hygiene and high-motivation–low-hygiene groups; i.e., the proportion with increasing ACE in the low-motivation–low-hygiene group was only half of that with decreasing ACE, whereas the reverse was the case in the high-motivation–low-hygiene group. Overall, the ACE of more than half of the PhD students either remained the same or decreased during their doctoral program, with only 42.95% of them reporting an increase.

**Table 6:** Association between profiles and ACE.

ACE	Low–low	Low–high	High–low	High–high
Sharp decrease	177 (27.30%)	134 (14.30%)	123 (8.20%)	64 (3.80%)
Slight decrease	135 (20.80%)	210 (22.40%)	233 (15.60%)	194 (11.70%)
No change	170 (26.20%)	279 (29.80%)	476 (31.90%)	509 (30.60%)
Slight increase	91 (14.00%)	181 (19.30%)	355 (23.80%)	422 (25.40%)
Sharp increase	76 (11.70%)	132 (14.10%)	305 (20.40%)	474 (28.50%)
*χ* ^2^	446.839***			

*N* = 4,740; ***p < 0.001.

## Conclusion and discussion

The main goal of this research was to identify the unobserved profiles of PhD-SS by focusing on their feelings about various aspects of the PhD training process. Using LPA, we identified four profiles, and referring to two-factor theory, we labeled them as *low-motivation–low-hygiene*, *low-motivation–high-hygiene*, *high-motivation–low-hygiene*, and *high-motivation–high-hygiene*, respectively. Also, we found that the different PhD-SS profiles were closely linked with the demographic characteristics of the PhD students and their changes in ACE.

This is the first empirical study to identify the profiles of PhD-SS using a person-centered approach. Our findings provide initial evidence supporting the heterogeneous characteristics of PhD-SS with various aspects of the doctoral training process. As mentioned above, four groups were found in this study according to the levels of satisfaction in different items, which means that the patterns of PhD-SS can typically be differentiated by the extent to which the training process satisfies students regardless of aspects. Most of the PhD students showed high satisfaction with academic-related factors (76.2% with academic cultivation and 86.1% with academic interaction). These findings do not categorically contradict previous research suggesting that Chinese PhD students have higher satisfaction in mentoring and competency development (Yuan and Li, 2017), indicating that the doctoral training process is at least rewarding in terms of the professional growth of PhD students. In contrast, hygiene factors were not well satisfied, as shown by the low satisfaction with life and financial support, which is highly consistent with the findings of Xiao et al. (2021).

After identifying the four-profile solution, we examined the associations between PhD student demographic characteristics and PhD-SS profiles. The logistic regression found that the four groups differed significantly in gender, age, work status, caring responsibilities, country, and study-abroad status.

When it comes to gender, female PhD students were less likely to be classified in the high-motivation groups. A possible reason for this is that women might be constrained by traditional social values (e.g., cultural expectations of subordinating to male authority) (Carter et al., 2013), leading to the academic path becoming rougher and bumpier for women. Existed studies have found that female PhD students are less likely to receive external funding (Hoffer et al., 2001) and become research assistants (Smith, 1995), and some female PhD students felt upset that they did not encounter a suitable mentor (Maher et al., 2004). All the aforementioned factors may contribute to lower satisfaction with the PhD training process among female students.

In terms of age, younger PhD students were more likely to be classified in the high-motivation–high-hygiene group. This may be because younger PhD students are less likely to be under pressure from financial issues, family responsibilities, etc. As the existing literature suggests, psychological stress has a negative impact on job satisfaction and life satisfaction (Brauchli et al., 2013), which echoes to some extent the differences in satisfaction categories regarding work status and caring responsibilities.

Regarding nationality, the probability of classifying PhD students in the high-motivation–high-hygiene group is significantly higher in the USA, UK, and Germany than in China. Lacking “the same breadth of externally funded scholarship programs as their counterparts have in the West” (Lam, 2011) may be an important reason for this difference. Finally, students studying for a PhD abroad were more likely to be satisfied with both motivation and hygiene factors, which is generally consistent with previous research (Harman, 2003).

Additionally, this study has provided some interesting results about the relationship between satisfaction profile and change in ACE in a population of PhD students. Previous research on early-stage scholars concluded that many factors—such as supervisors and economics—have a great impact on students’ decisions about whether to pursue a lifelong academic career (Barrett and Barrett, 2011; Sauermann and Stephan, 2012; Pilbeam et al., 2013; Roumell et al., 2014), which is also supported by the present study. Furthermore, we find that increasing or decreasing ACE is related more closely to motivation factors among PhD students. Even if the hygiene factors are not well satisfied, PhD students may still hold higher ACE if they have higher satisfaction with motivation factors. Conversely, PhD students with higher satisfaction with hygiene factors but lower satisfaction with motivation factors may lose enthusiasm for academia. That is, in the case of maintaining and stimulating ACE, motivation factors can compensate for the negative impact of the absence of hygiene factors, not the other way around. Therefore, it can be seen that two-factor theory has a certain explanatory power for changes in ACE, but it must be adjusted in a certain way considering the special characteristics of the population.

## Contributions and limitations

The theoretical and practical value of this study is reflected mainly in the following. First, four subcategories of PhD-SS were identified by adopting a latent profile analysis, which preserves individual integrity, leading to a more accurate assessment of the students’ feelings about the training process. Second, this study examined the differences in satisfaction in terms of the demographic characteristics of each category, thereby enriching the knowledge about the antecedents of PhD-SS. Finally, the significant influence of the training process on PhD students’ ACE has also been revealed. To some extent, this study has also verified the application value of Herzberg’s two-factor theory in motivating PhD students’ ACE.

However, this study still has some limitations. For example, the questionnaire was collected mainly from PhD students who were studying science and technology, so we must be cautious when extending the results to other disciplines; future studies with various PhD student datasets from different disciplines or specialties are needed before drawing general conclusions about different population groups. Also, this study used cross-sectional data from a self-reported technique to identify PhD-SS profiles, and we only compared the proportion of PhD students in different profiles in terms of increasing or decreasing ACE; we cannot make a causal inference about the relationship between PhD-SS and ACE. So, future research could conduct a longitudinal study to find the changes in ACE of PhD candidates at various stages of the PhD program and further find a causal link between these two variables.

## Data availability statement

The original contributions presented in the study are included in the article/supplementary material, further inquiries can be directed to the corresponding author.

## Ethics statement

Ethical review and approval was not required for the study on human participants in accordance with the local legislation and institutional requirements. Written informed consent from the (patients/ participants OR patients/participants legal guardian/next of kin) was not required to participate in this study in accordance with the national legislation and the institutional requirements.

## Author contributions

YY: conception, conceptualization, theoretical direction, interpretation of the statistical analyses and results, and original draft preparation. JC: supervision and the writing-reviewing of this project. All authors contributed to the article and approved the submitted version.

## Funding

This study was supported by the Social Science Foundation of Hunan province (18YBQ092).

## Conflict of interest

The authors declare that the research was conducted in the absence of any commercial or financial relationships that could be construed as a potential conflict of interest.

## Publisher’s note

All claims expressed in this article are solely those of the authors and do not necessarily represent those of their affiliated organizations, or those of the publisher, the editors and the reviewers. Any product that may be evaluated in this article, or claim that may be made by its manufacturer, is not guaranteed or endorsed by the publisher.
